# Differential expression of proteomics models of colorectal cancer, colorectal benign disease and healthy controls

**DOI:** 10.1186/1477-5956-8-16

**Published:** 2010-03-25

**Authors:** Ming Liu, Chun-Feng Li, Hong-Sheng Chen, Luo-Qiang Lin, Chun-Peng Zhang, Jin-Lu Zhao, Yan Liu, Shu-Jun Zhang, Jun-Chao Jin, Lei Wang, Jia-Ren Liu

**Affiliations:** 1 Treatment Center of Oncology, the Fourth Affiliated Hospital of Harbin Medical University, Harbin, 150001, PR China; 2 Department of Abdominal Surgery, the Affiliated Tumor Hospital of Harbin Medical University, Harbin, 150081, PR China; 3 Public Health College, Harbin Medical University, Harbin, 150081, PR China; 4 Current address: Harvard Medical School, 300 Longwood Ave, Boston, MA, USA

## Abstract

**Background:**

Colorectal cancer (CRC) is often diagnosed at a late stage with concomitant poor prognosis. The hypersensitive analytical technique of proteomics can detect molecular changes before the tumor is palpable. The surface-enhanced laser desorption/ionization-time of flight-mass spectra (SELDI-TOF-MS) is a newly-developed technique of evaluating protein separation in recent years. The protein chips have established the expression of tumor protein in the serum specimens and become the newly discovered markers for tumor diagnosis. The objective of this study was to find new markers of the diagnosis among groups of CRC, colorectal benign diseases (CBD) and healthy controls. The assay of SELDI-TOF-MS with analytical technique of protein-chip bioinformatics was used to detect the expression of protein mass peaks in the sera of patients or controls. One hundred serum samples, including 52 cases of colorectal cancer, 27 cases of colorectal benign disease, and 21 cases of healthy controls, were examined by SELDI-TOF-MS with WCX2 protein-chips.

**Results:**

The diagnostic models (I, II and III) were setup by analyzed the data and sieved markers using Ciphergen - Protein-Chip-Software 5.1. These models were combined with 3 protein mass peaks to discriminate CRC, CBD, and healthy controls. The accuracy, the sensitivity and the particularity of cross verification of these models are all highly over 80%.

**Conclusions:**

The SELDI-TOF-MS is a useful tool to help diagnose colorectal cancer, especially during the early stage. However, identification of the significantly differentiated proteins needs further study.

## Background

Colon cancer is one of the most common cancers and the fourth leading death in the malignant tumors in the world. It is reported that approximately 106,100 new cases of cancer would be diagnosed, and more than 49,920 people would die from cancer in the United States alone in 2009 [1]. The occurrence of colorectal cancer was regarded as a multigenic disease according to modern molecular biology, and genetic abnormality plays a critical role in the development and progression of cancer cells [2,3]. By now, except for chemoprevention, there are no certain ways proven to be benefited for preventing colon cancer. There is an urgent need for methods to predict and diagnose the patients in the early stage of colorectal cancer. Therefore, looking for new techniques with validly, highly and powerful sensitivity are very important for the prevention, prognosis, and treatment of colorectal cancer. The proteomics have very important contribution to the cancer diagnosis based on valuable information of the pathologic physiology of the tumor as well as finding new antitumor drugs [4]. The proteomic pattern would facilitate the early detection and the development of tumor biomarkers as well as therapeutic efficacy anticancer drugs [5].

The multichannel detection capability of mass spectrometry (MS) enables the position sensitive analysis of hundreds of different molecules in a single experiment. MS is increasingly used to profile the serum peptidome [6]. Magnetic bead-assisted serum peptide capture coupled to matrix assisted laser desorption/ionization time-of-flight MS (MALDI-TOF-MS), a novel non-electrophoresis-based proteomic technology, is a serum peptide profiling strategy gaining in popularity compared to surface-enhanced laser desorption/ionization (SELDI) - based platforms due to superior resolution of MALDI instruments. The MALDI-TOF-MS is also a possibility to obtain structural (MS/MS) information of signature peptides and superior binding capacity of the magnetic beads compared to a flat SELDI-chip surface [7]. It has been shown to be useful in the discovery of potential diagnostic markers for cancers such as prostate [8], ovarian [9], hepatic [10], and breast cancer [11]. In a previous study, the urine proteome as the early detection of colorectal cancer from colorectal cancer patients was examined by a SELDI method [12]. In another study, the serum proteome from patients of colorectal cancer, benign colorectal diseases and healthy volunteers was also detected by SELDI-TOF-MS. The four proteins were regarded as effective biomarkers for diagnostics and therapeutic strategies or monitoring micrometastasis [13]. Thus, this system is a novel, extremely sensitive, and rapid method to analyze complex mixtures of proteins and peptides. The objective of the present study was to determine whether comprehensive proteomic profiling of serum coupled with bioinformatic analysis methods originally designed for gene expression data could identify a proteomic printing for effectively differentiating colorectal cancer or benign disease patients.

## Methods

### Patient and Control Sources

One hundred patients or controls were chosen from the Affiliated Tumor Hospital of Harbin Medical University, P. R. China between February and July 2004. There were 52 cases with colorectal carcinoma (CRC) (28 males and 24 females) from 30 to 80 years old (average 58.9 ± 13.4) and 27 cases (from 43 to 69 and average 55.1 ± 8.6 years old) with colorectal benign disease (CBD) which were pathologically diagnosed after surgery (11 males and 17 females) from clinic diagnosis. All patients did not receive any therapy before blood collection. All patients with CRC were separated to I, II and III stages according Dukes' standards and these cases did not have distant metastasis. Twenty-one healthy volunteers (11 males and 10 females) as healthy controls from 30 to 71 years old (average 47.2 ± 5.8) were selected from the staffs who were working at clinic. All patients and healthy controls thoroughly agreed with and signed the agreements consent for the investigation in accordance with the ethical guidelines of Harbin Medical School Ethical Committee. The sera from patients or healthy controls were distributed into 500 μL aliquots and stored frozen at -80°C for serum proteomic analysis.

### Reagent and Instrument

Experiments were performed using SELDI-TOF-MS instrument, chip WCX2, and the corresponding analytical software of Ciphergen-Protein-Chipsoftware 5.1 (Ciphergen Biosystems Inc, Fremont, CA). The reagents such as acetonitrile (HPLC grade), trifluoroacetic acid (TFA), sodium acetate (250 g), SPA ground substance, CHAPS, TRIS-HCL, DL-dithiothreitol (DTT) and urea, were bought from the Sigma-Aldrich Company (St. Louis, MO).

### Sample Preparation

The serum samples from the experimental or control group were centrifuged at 10,000 rpm for 5 min at 4°C. Ten μL of the serum sample was filled with 20 μL of 9 U balanced solutions (9 mol/L Ureas, 2% CHAPSs, 50 mmol/L Tris-HCL, pH 9.0, and 1% DTTs) into the bores with shaking. The samples were shaking with ice bath (MS1 Minishaker) at a rate of 400 - 600 rpm for 30 min and then added 360 μL of natrium aceticum buffer (50 mmol/L NaAc, pH 4.0) with shaking.

### Pretreatment, Application of Sample, and Elution

The WCX2 chip (Ciphergen Biosystems Inc, Fremont, CA) was used throughout this study because this chip could distinguish the weak differential peaks. The WCX2 chip placed into the bio-processor was filled each bore with 200 μL of natrium acetic buffer and spun the bio-processor at a rate of 400-600 rpm for 5 min and then the buffer was removed. The same process mentioned above was repeated again. Each bore of the bio-processor was filled with 100 μL of the sample, agitated at a rate of 400-600 rpm for 1 h at 4°C (ice bath). After removing the sample, 200 μL sodium acetate buffer (50 mmol/L NaAc, pH 4.0 or the binding buffer in kit) was added to each bore, and was spun at a rate of 400 - 600 rpm for 5 min at room temperature. This process was also repeated again. Subsequently, 200 μL of HPLC flow phase was added to each bore, and then discarded immediately. This procedure was repeated twice. The chip was taken out and added 0.5 μL of SPA solution (50% CANs + 0.5% TFAs) to each well after exsiccation. After sample exsiccation, SPA was added again. The samples were dried and analyzed by the SELDI-TOF-MS system.

### Chip Examination, Data Acquisition and Parameter Enactment

Chips were placed in the SELDI-TOF-MS system (Ciphergen Biosystems Inc, Fremont, CA), and time-of-flight spectra were generated by averaging 192 laser shots collected in the positive mode at laser intensity 215, detector sensitivity 7. The mass range from molecular weight 10,000 - 20,000 Da or the highest 50,000 Da was selected for analysis because this range contained the majority of the resolved protein/peptides. The range of data collection was designed from 10,000 to 50,000 *m/z *(mass-to-charge).

### Statistical analysis

The data were analyzed by software of Ciphergen-Protein-Chip-software 5.1. When the differentiated expressions of protein mass peak were found among the groups of colorectal cancer, colorectal benign disease and healthy controls, these data in the Excel format were imported into the software of Ciphergen-Protein-Chip-software 5.1. The significantly different expression of protein mass peaks (*P *< 0.05) was listed by the software. Subsequently, the differentiated expressions of protein mass peak were analyzed by discriminatory analysis. The best alignment combination was analyzed by Biomarker Wizard 3.1. Each serum sample was performed at least in triplicate to confirm reproducibility and reduce bias.

## Results

The protein mass peaks (*m/z*) were sieved with the s/n exceeding 2 or 5. More than 10% of *m/z *was sieved in simultaneous samples with the discrepancy of identical spinnacle in different samples lower than 0.3% after removing the noise of initial data. One hundred and eight-five significant protein mass peaks were found from 2000 to 20,000 peaks of m/z between the colorectal cancer and control groups, 139 protein mass peaks between the CRC and CBD groups and 139 protein mass peaks between the CBD and healthy control groups.

**Figure 1:**
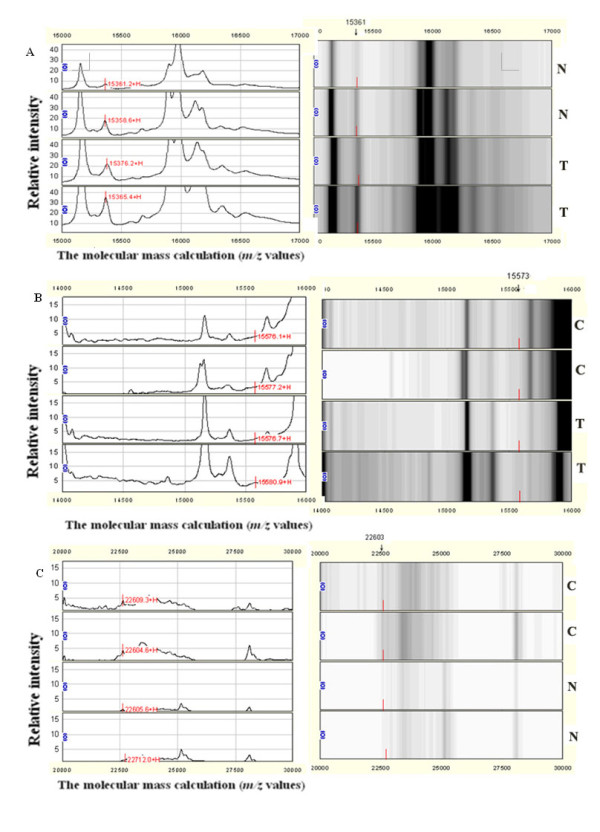
**Protein profiling on WCX2 chips**. Representative overview of protein profiling on WCX2 chips showing spectral map (left panel) and gel view (right panel) of the serum samples. SELDI analysis of human serum for proteomic pattern in the colorectal benign disease (T), healthy control (N) and colorectal cancer (C) samples with mass spectra (left) and gel view (right). Differentially expressed proteins were found in *m*/*z *values of (A) 15361 Da, (B) 15573 Da and (C) 22603 Da.

**Figure 2:**
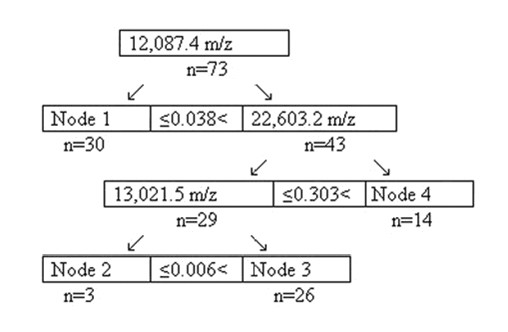
**Discrimination decision tree models of serum protein mass-spectrum between the CDR and the healthy controls**. The "n" is the number of samples; the node is a final node.

After discriminatory analysis, 3 of 185 protein mass peaks were chosen by optimization to establish the combined diagnostic model I (Table 1 and Figure 1), the categorizing decision tree was built up, and 4 final crunodes were determined (Figure 2). As shown in Table 1, three protein mass peaks were 12,087.4, 22,603.2, and 13,021.5 m/z (Table 1). The accuracy of diagnostic model I was 87.67% (64/73), with a sensitivity of 86.54% (45/52) and specificity of 90.48% (19/21); and the accuracy of crossing verification was 82.19% (60/73), with the sensitivity of 80.77% (42/52) and specificity of 85.71% (18/21).

**Table 1:** The comparison of 3 protein mass peaks between the colorectal cancer (CRC) and healthy controls (HC) groups (mean ± S.D.)

Protein mass-peak(m/z)	The CRC	The HC	*P*
12087.4	0.044 ± 0.063	0.080 ± 0.045	0.005
22603.2	0.292 ± 0.207	0.182 ± 0.104	0.010
13021.5	0.032 ± 0.021	0.019 ± 0.011	0.022

**Figure 3:**
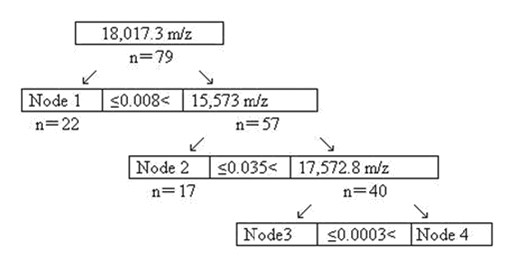
**Discrimination decision tree models of serum protein mass-spectrum between the CRC and CBD groups**. "n" is the number of the samples, and the node is the final node.

Three of one hundred and thirty nine protein mass peaks between the CRC and CBD groups were also chosen by optimization to setup the combined diagnostic model II (Table 2 and Figure 1), build up the categorizing decision tree and get 4 final crunodes (Figure 3). Three protein mass peaks (17,572.8, 15,573, and 18,017.3 m/z) are shown in Table 2. The accuracy of diagnostic model II was 88.61% (70/79), with the sensitivity of 86.54% (45/52) and the specificity of 92.59% (25/27), and the accuracy of crossing verification was 87.34% (69/79), with the sensitivity of 86.54% (45/52) and the specificity of 88.89% (24/27).

**Table 2:** The comparison of 3 protein mass peaks between the colorectal cancer (CRC) and colorectal benign disease (CBD) groups (mean ± *S.D*.)

Protein mass-peak(m/z)	The CRC	The CBD	*P*
17572.8	0.060 ± 0.043	0.055 ± 0.029	0.003
15573.0	0.027 ± 0.029	0.015 ± 0.010	0.059
18017.3	0.035 ± 0.053	0.053 ± 0.044	0.010

Another 139 significant protein mass peaks expressed differently were analyzed between the CBD and healthy control groups. The protein mass peaks of 15,361, 17,389.7, and 14,501.8 m/z were chosen by optimization (Table 3). The combined diagnostic model III was also setup. The accuracy of this model was 97.92% (47/48), with the sensitivity of 100% (27/27) and the specificity of 95.24% (20/21), and the accuracy of crossing verification was 91.67% (44/48), with the sensitivity of 92.59% (25/27) and specificity of 90.48% (19/21).

**Table 3:** The comparison of 3 protein mass peaks between the groups of colorectal benign disease (CBD) and healthy controls (HC) (mean ± S.D.)

Protein mass-peak(m/z)	The CBD	The HC	*P*
15361.0	0.810 ± 0.799	0.479 ± 0.346	0.005
17389.7	0.045 ± 0.030	0.022 ± 0.024	0.009
14501.8	0.192 ± 0.083	0.138 ± 0.068	0.046

## Discussion

The hypersensitive analytical technique of proteomics can detect molecular changes before the tumor is palpable. This technique has an important role in the diagnosis and monitoring of tumors. SELDI-TOF-MS is a newly-developed technique of evaluating protein separation in recent years. The protein chips have established the expression of tumor protein in the serum specimens including breast, prostate, and bladder cancer. Some of the proteins from chips have become the newly discovered markers for tumor diagnosis, with higher sensitivity and specificity than the former markers [8,14-18]. There are many noninvasive diagnostic methods of colorectal cancer such as the serum tumor markers (CEA, TPA, and CA199, etc.), the fecal occult blood test, biochemistry, and immunologic test. However, there are high rates of false positives and false negatives. The sensitivity and specificity of serum tumor markers still go back and forth from 50 to 70% [19].

In a previous study [12], the assays of MALDI and SELDI were used to detect the samples of urine from 67 patients with CRC and 72 non-cancer controls. The intensities of 19 peaks that differed significantly between cancer and non-cancer patients were found by multiple linear regressions. Logistic regression classifiers based on peak intensities identified CRC with up to 78% sensitivity at 87% specificity. Zheng, et al. [13] reported that the serum proteome from 63 patients with colorectal cancer, 20 patients with CBD and 26 healthy volunteers was also determined by a SELDI-TOF-MS assay. The two peaks (2753.8 and 4172.4 m/z) detected in that study have the potential for assistance in diagnostics and therapeutic strategies in colorectal cancer and the two proteins (9184.4 and 9340.9 m/z) were effective biomarkers for monitoring micrometastasis. In another study [20], three serum proteins of diagnostic potential (complement C3a des-arg, *α*1-antitrypsin and transferring) were identified by SELDI from 62 CRC patients and 31 noncancer subjects. In our study, three serum protein mass peaks (12,087.4, 22,603.2, and 13,021.5 m/z) from 185 significantly different protein mass peaks between CRC and control groups were found and established the combined diagnostic model I. The accuracy of this model was 87.67%, with a sensitivity of 86.54% and specificity of 90.48%. Simultaneously, the combined diagnostic models II, III were also setup based on 3 serum protein mass peaks among the CRC, benign disease and healthy control groups. However, these differentiated proteins are needed to identify using the assays of synthetic stable isotope peptides or ELISA and to further confirm these combined diagnostic models using the patients with CRC. We also need to increase the cases of early stage of CRC in the analysis, in order to increase the sensitivity and specificity of combined diagnostic models.

The SELDI-TOF-MS and protein chip technique could discriminate between patients with and without tumors. However, there are limitations in SELDI-TOF MS whole serum proteomic profiling with IMAC surface to specifically detecting colorectal cancer [21]. Wang, et al. [21] generated a classifier consisting of two serum protein mass peaks (3961 and 5200 *m*/*z*) that distinguished 154 patients with CRC from 67 non-cancerous controls, with promising diagnosis efficiency. But these two peaks were not CRC-specific; they could not separate CRC from other cancer types in the case of patients who had two or more types of cancers. Thus, whether we need increase protein mass peaks (least 3) and add the known markers in the combined diagnostic models, these efforts are underway in ongoing studies.

## Conclusion

In summary, our study indicates that the SELDI-TOF-MS technique has instructional contributions to diagnosis of colorectal cancer, especially in early diagnosis, preoperative treatment, staging and prognosis. Our findings have potential contribution of extensive survey-aided detection in time among the high-risk patients with CBD. However, significantly differentiated proteins need to be identified. A further study is needed to improve the sensitivity and specificity of combined diagnostic models.

## Competing interests

The authors declare that they have no competing interests.

## Authors' contributions

ML conceived the idea of proteomics study, participated in its design and coordination, and performed major portion of the sample analysis. CFL and HSL did SDS-PAGE, performed the gel slicing and LC/MS of peptides in the analysis of the proteomics data. LQL and CPZ collected the cases of patient and healthy control. JLZ and YL participated in blood samples collection from patient and healthy control. SJZ diagnosed types of colorectal pathology. JCJ and LW performed the proteomic analysis of the samples. JRL reviewed the design plan, analyzed data, drafted and revised manuscript. All authors read and approved the final manuscript.
